# Lay Evaluation of Financial Experts: The Action Advice Effect and Confirmation Bias

**DOI:** 10.3389/fpsyg.2016.01476

**Published:** 2016-09-27

**Authors:** Tomasz Zaleskiewicz, Agata Gasiorowska, Katarzyna Stasiuk, Renata Maksymiuk, Yoram Bar-Tal

**Affiliations:** ^1^Faculty in Wroclaw, SWPS University of Social Sciences and HumanitiesWroclaw, Poland; ^2^Institute of Psychology, Maria Curie–Sklodowska UniversityLublin, Poland; ^3^Sackler Faculty of Medicine, Tel Aviv UniversityTel Aviv, Israel

**Keywords:** financial advice, epistemic authority, action advice effect, confirmation bias, judgment, motivation, financial services

## Abstract

The goal of this experimental project was to investigate lay peoples’ perceptions of epistemic authority (EA) in the field of finance. EA is defined as the extent to which a source of information is treated as evidence for judgments independently of its objective expertise and based on subjective beliefs. Previous research suggested that EA evaluations are biased and that lay people tend to ascribe higher EA to experts who advise action (in the case of medical experts) or confirm clients’ expectations (in the case of politicians). However, there has been no research into biases in lay evaluations of financial experts and this project is aimed to fill this gap. Experiment 1 showed that lay people tended to ascribe greater authority to financial consultants who gave more active advice to clients considering taking out a mortgage. Experiment 2 confirmed the action advice effect found in Experiment 1. However, the outcomes of Experiments 2 and – particularly – 3 suggested that this bias might also be due to clients’ desire to confirm their own opinions. Experiment 2 showed that the action advice effect was moderated by clients’ own opinions on taking loans. Lay people ascribed the greatest EA to the advisor in the scenario in which he advised taking action and where this coincided with the client’s positive opinion on the advisability of taking out a loan. In Experiment 3 only participants with a positive opinion on the financial product ascribed greater authority to experts who recommended it; participants whose opinion was negative tended to rate consultants who advised rejecting the product more highly. To conclude, these three experiments revealed that lay people ascribe higher EA to financial consultants who advise action rather than maintenance of the *status quo*, but this effect is limited by confirmation bias: when the client’s *a priori* opinion is salient, greater authority is ascribed to experts whose advice confirms it. In this sense, results presented in the present paper suggest that the action advice effect might be also interpreted as a specific manifestation of confirmation bias.

## Introduction

Bernard Madoff became one of the dark symbols of the recent global financial crisis. In 2009 he pleaded guilty to creating one of the biggest investment frauds in the history of international finance and was sentenced to 150 years in prison. His wealth management business, which was in fact a massive Ponzi scheme (Frankel, 2012), defrauded 10s of 1000s of investors of billions of dollars. Madoff’s clients included not just individual investors, but also banks, hedge funds, and charities (McCool and Graybow, 2009). One of the questions asked by journalists and market commentators was how could Madoff have been so successful in deceiving so many people? Why did so many individual and institutional investors recognize him as an expert? Why were people ready to trust him and follow his recommendations? These questions are important to the Madoff case but might also be posed in more general terms. Why and when are consumers willing to believe an individual has financial expertise and to entrust financial advisors with their money – sometimes their life savings? What characteristics make financial advisors more competent and trustworthy in the eyes of their clients? The aim of the research presented in this paper was to find answers to these questions, which are of practical as well as theoretical importance. The data we collected in a series of three experiments suggest that lay people tend to judge financial consultants as more competent when they advice action rather than inaction and when their recommendations confirm lay people’s naïve opinions.

Lay perceptions of expert knowledge have been considered from a theoretical point of view in the lay epistemic theory proposed by Kruglanski (1989, 2012). Lay epistemic theory introduced the concept of epistemic authority (EA), defining it as “the extent to which an individual is inclined to treat a source’s information as incontrovertible evidence for her or his judgment” (Kruglanski, 2012, p. 212). The term EA is used to refer to subjective beliefs about a source of knowledge or expertise. Sources can acquire EA to the extent that an individual believes that they possess characteristics that give them such authority (Raviv et al., 1993). In other words, EA comprises the level of knowledge which individuals attribute to the source, the degree to which they trust the source’s knowledge, are willing to change their opinions under the influence of the source, and are willing to change their behavior under the influence of the source (Raviv et al., 1993).

The characteristics that are used to identify a source as an EA can be general – e.g., a professional or social role (e.g., leader or physician), level of education (e.g., holder of a doctorate), an appearance in print (e.g., in a book or a newspaper) – or specific, as when EA is assigned to a particular person, or to a particular newspaper (Kruglanski et al., 2005).

The concept of EA is akin to the notion of source credibility, which refers to a conjunction of perceived expertise and trustworthiness. EA can override other types of information and exert a determining influence on individuals’ opinions and behavior. They process the information from a source with EA more extensively, are more certain of it, and tend to act in accordance with its implications. In the Lay Epistemology framework, EA functions as a ‘stopping mechanism.’ It effects cessation of the hypothesis generation sequence and crystallization of confident knowledge (Kruglanski et al., 2005; Kruglanski, 2012). Individuals may be willing to follow the financial advice of people perceived as having EA without testing it or considering alternatives; they may also be more confident of decisions based on recommendations given by experts with high EA (Kruglanski et al., 2010).

The importance of EA in knowledge or judgment formation induced researchers to try finding out what factors lend a source EA. Recent research in health psychology has demonstrated that patients attributed greater EA to physicians who recommended an active treatment (inoculation or prenatal genetic tests) over those who advised against it (i.e., recommended maintaining the *status quo*) or gave no recommendation at all (Barnoy et al., 2009, 2012; Bar-Tal et al., 2013; Stasiuk et al., 2016). Stasiuk et al. (2016) concluded that people might be biased when judging the level of expertise of their physicians, such that physicians who recommend more active treatment are judged to have greater medical EA. The robustness of the phenomenon they observed motivated the authors to wonder whether this bias exists also in other areas where lay people have to judge the expertise of an advisor (e.g., in the economic, political, or legal domain). Should this be the case, then advising a more active approach might be a prerequisite for being accorded high EA, and serve as a kind of a universal heuristic (action – authority; Stasiuk et al., 2016). Following this suggestion, we conducted a series of experiments to examine the universal nature of the action advice effect and to test whether advising action rather than inaction positively affects the evaluation of experts also in the domain of personal finance. In the current research we will use the term ‘action advice’ to refer to advice to do something or to act; this can be contrasted with ‘inaction advice,’ advice to do nothing or to maintain the *status quo*. In the field of health, action advice is associated with more aggressive treatment and more invasive medical intervention, whereas in the financial domain it might involve taking greater risk, taking a larger loan or just investing more money.

Interestingly, the ‘activity level’ of advice is not the only source of bias in evaluating experts’ EA. Research conducted on evaluations of political expertise has shown that similarity of political views might be particularly important to evaluations of experts’ EA. Raviv et al. (1993) reported that individuals assigned greater EA to political leaders who shared their own opinions. This suggests that lay evaluations of experts’ authority depends not only on whether they recommend a more active course but also whether their advice is consistent with clients’ *a priori* expectations or opinions. The existence of the latter effect would imply that confirmation bias is important to perceptions of an expert’s authority.

To summarize, two psychological factors seem to influence lay perceptions of experts’ authority: (1) the extent to which they advise an active approach and (2) the extent to which their advice confirms the client’s prior opinions. Research to date suggests that lay evaluations of EA might be biased in favor of advisors proposing larger interventions or offering advice which is consistent with what the client expects from an expert. We use the term ‘bias’ here because we assume that the impact of the above two factors is independent of objective determinants of expertise (e.g., an expert’s experience in the field or the correctness of her advice in the past).

Despite the fact that many empirical studies have been carried out to test the assumptions of lay epistemic theory (Kruglanski et al., 2005, 2010; Kruglanski, 2012) no study has directly examined how the theory applies to lay judgments on financial issues. Moreover, these two effects – the action advice effect and confirmation bias – were investigated separately for two different domains (medical and political). Previous research has not tested how these effects interplay with each other and whether one of them (the action advice bias) might be limited by the other (the confirmation bias). Our study aimed to fill this gap and investigate how two psychological effects of interest influence lay evaluations of the authority of financial consultants. We chose the domain of finance to test our hypotheses because consumers who search for advice from financial consultants do not have access to clear cues informing about the experts’ competence. In other words, it is not obvious which factors inform about the level of knowledge possessed by a financial advisor. On the contrary, when patients have to judge the authority of physicians, they might base their evaluations on such evidence as professional title, academic degree or the place in the organizational hierarchy. Therefore, the evaluation of EA in the financial domain seems to depend to a higher degree on vague aspects and, as a consequence, be more susceptible to biases such as action advice bias or confirmation bias.

### Perception of Epistemic Authority in the Financial Domain

Although, information about how people decide who should be considered an expert has important implications for information exchange between lay persons and experts, to the best of our knowledge no study in cognitive or social psychology has examined this question directly. Many research programs have investigated determinants of the correctness of economic expertise and revealed cognitive errors and biases that might limit the quality of financial advice (Camerer and Johnson, 1991; Shanteau, 1992; Zaleskiewicz, 2011). However, little is known about how people who are receivers of experts’ recommendations evaluate sources of financial expertise and how they rate their reliability. Studying lay perceptions of financial advice would significantly contribute to a better understanding of the psychological nature of the interaction between advisors and their clients.

In the contemporary world people are forced to make choices about financial activities as saving, insurance, taking out bank loans, currency exchange and real estate transactions. To make good choices about such activities (i.e., to make timely decisions and choose appropriate financial instruments) individuals usually have to turn to various sources of expert knowledge to acquire or verify information. Understanding the biases affecting people’s trust in experts’ financial knowledge is therefore highly relevant to understanding consumers’ perceptions of the authority of financial or investment consultants. For example, it may be the case that an investment advisor’s recommendation to invest more money in a given stock (action advice) rather than to avoid trading (inaction advice) is likely to enhance the potential investor’s perception of the EA of the advisor. A similar boost to the advisor’s authority might result from a recommendation which confirms the client in his or her preferred approach or opinion (e.g., a client who tends to take large financial risks might accord greater authority to an advisor who recommends investing in risky stocks).

The main aim of this research was to examine the existence of bias in attributions of expertise and consider possible sources of such bias in the field of the financial expertise. Specifically, we analyzed how the extent to which an active rather than passive approach is advised affects the evaluation of expert’s EA and investigated the extent to which the action advice effect is limited by the confirmation bias.

## Experiment 1: The Effect of a Recommendation for ‘Action’ on Lay Evaluations of Expert’s Epistemic Authority

As we have pointed out earlier in this paper, people attribute greater EA to physicians who recommend any form of treatment than to those who advise to wait (Barnoy et al., 2012; Bar-Tal et al., 2013; Stasiuk et al., 2016). The main goal of the first experiment was to test whether the same effect applies to evaluations of financial experts. In particular, we hypothesized that lay people considering whether to take a bank loan (mortgage) would assign greater authority to financial advisors who advised action (i.e., taking on the debt) rather than those who advised against this (the more passive approach). We also manipulated experience, a more objective source of expertise, as we hypothesized that greater EA would be attributed to advisors with more experience. We used a mortgage as an example of a financial product because in Poland one of the most common reasons for meeting a financial advisor is to discuss a mortgage.

### Method

#### Participants

We recruited 144 employed Polish adults having their own income (*M*_age_ = 29.18, *SD* = 8.57; 97 women). The reason for selecting this group was to avoid participants who do not earn money and have little experience with making financial decisions (e.g., younger students). The experiment was conducted online. The study was voluntary, anonymous and in agreement with the guidelines of the Ethical Committee of the SWPS University of Social Sciences and Humanities, Faculty in Wroclaw. Participants did not receive any compensation. All materials and interactions with participants were in Polish language.

#### Procedure and Measures

Participants were randomly assigned to one of the eight conditions in a between-subjects design, with type of advice (four levels) and advisor experience (two levels) as independent variables. After providing personal information (age; gender) and giving the informed consent, participants were asked to read a hypothetical ‘loan’ scenario. Participants were asked to imagine that their friend and his wife were renting a flat and had been considering for some time whether to take out a mortgage to buy a flat or carrying on renting and had therefore visited the bank to consult a financial advisor^1^. Next, they were given information about the advice offered by the hypothetical consultant in order to test if their evaluations of advisors’ EA would depend on whether advisors recommended action or inaction. However, instead of using only two manipulation levels (action vs. inaction) we introduced four levels described above to better imitate real life interactions between consultants and their clients. It seems that in real life advisors’ recommendations refer to specific rather than more general courses of action. The advice depended on the experimental condition: (1) ‘against’ – the consultant advised the couple against taking out a mortgage and suggested that they should continue to rent; (2) ‘postpone’ – the consultant advised the couple to wait half a year and monitor the market; (3) ‘small action’ – the consultant advised taking out as small a loan as possible; (4) ‘large action’ – the consultant advised taking out a loan large enough to buy a flat and all the requisite home appliances.

Finally, the scenario gave information about the advisor’s experience. Depending on the experimental condition, the advisor was described as having either 15 years or just 1 year of experience as a financial consultant.

#### Dependent Measures

After reading the scenario participants were asked to fill in the short version of the EA scale to provide a measure of the EA attributed to the advisor in the scenario. The EA scale was originally developed and validated by Raviv et al. (1993) on the basis of the EA concept introduced by Kruglanski (1989). The scale was widely used by other researchers to study EA in different domains (e.g., Raviv et al., 2003; Kruglanski et al., 2005; Madar and Bar-Tal, 2009; Barnoy et al., 2011, 2012; Bar-Tal et al., 2013). The shortened version of the questionnaire that we used in the present project was adapted from Barnoy et al. (2012) and consisted of six items referring to the degree of knowledge which the participants attributed to the advisor and the degree to which the participants trusted the advisor’s knowledge (‘To what extent do you think the advisor is an expert in finance?,’ ‘To what extent are this advisor’s arguments based on verified knowledge?,’ ‘To what extent do you accept what this advisor says as correct?,’ ‘To what extent do you think that other advisors would say the same?,’ ‘To what extent do you trust this advisor?,’ ‘To what extent is this advisor a reliable source of information?’; Cronbach’s α = 0.91). Responses were given on a six-point scale ranging from 1 = ‘definitely disagree’ to 6 = ‘definitely agree.’

#### Auxiliary Measures (Manipulation Check)

Participants were also asked to respond to six additional items that were included as a manipulation check to make sure that the participants had distinguished between the different recommendations (‘The advice was definitely against taking the loan,’ ‘The advice was definitely to take the loan,’ ‘The advisor’s opinion on taking the loan was positive,’ ‘The advisor’s opinion on taking the loan was negative,’ ‘The advisor recommended taking a small loan,’ and ‘The advisor recommended taking a large loan’). Responses were given on a six-point scale ranging from 1 = ‘definitely not to 6 = ‘definitely yes.’

### Results

#### Manipulation Check

Before testing the main research hypotheses, we performed a manipulation check based on 4 × 2 MANOVA with the six items described in Section “Auxiliary Measures (Manipulation Check).” We observed a main effect of advice, Wilks λ = 0.12, *F*(18,371) = 22.99, *p* < 0.001, $\eta_{p}^{2}$ = 0.51, but there was no main effect of experience, Wilks λ = 0.98, *F*(6,131) = 0.47, *p* = 0.83, and no interaction between experience and advice, Wilks λ = 0.88, *F*(18,371) = 0.90, *p* = 0.53. Further planned comparisons were carried out to test the correctness of people’s responses to specific items. These comparisons revealed that participants were able to distinguish between the four types of advice, thus confirming the effectiveness of the experimental manipulation. Detailed results are presented in Supplementary Table S1. We also calculated a new variable indicating the number of correct answers to the manipulation check items. This variable ranged from 0 – answers to all items were incorrect, to 6 – answers to all items were correct. None of the participants scored 0 on this variable, while 94.44% of them answered correctly to four or more items used as the manipulation check.

#### Advisor’s Epistemic Authority

Two-factor ANOVA revealed main effects of both putatively EA-related independent variables, namely type of advice, *F*(3,136) = 7.33, *p* < 0.001, η^2^ = 0.14, and experience, *F*(1,136) = 8.26, *p* = 0.005, η^2^ = 0.06. The interaction between these two factors was not significant, *F*(3,136) = 0.31, *p* = 0.81.

**FIGURE 1:**
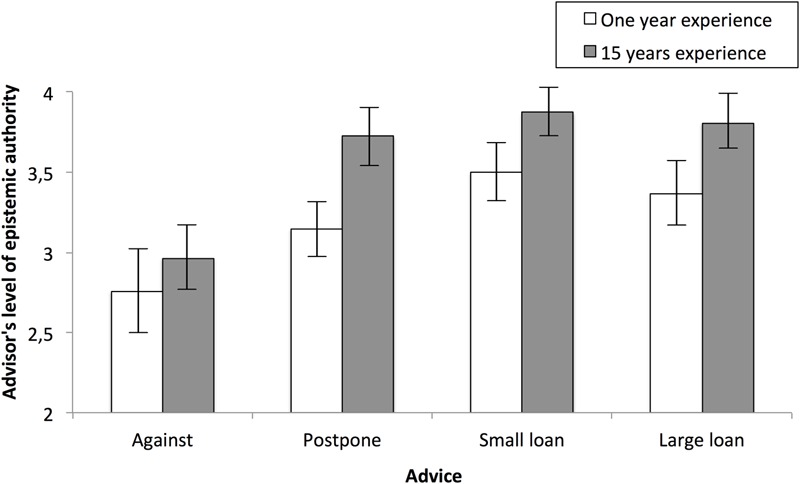
**Ascriptions of epistemic authority as a function of advisor’s experience and type of advice**.

Planned comparisons showed that the ‘against’ advice yielded lower ratings of advisor EA (*M* = 2.85, *SD* = 1.01) than the other three types of advice [‘postpone’: *M* = 3.44, *SD* = 0.80, *F*(1,140) = 8.48, *p =* 0.004, η^2^ = 0.06; ‘small loan’: *M* = 3.69, *SD* = 0.70, *F*(1,140) = 17.34, *p* < 0.001, η^2^ = 0.11; ‘large loan’: *M* = 3.59, *SD* = 0.90; *F*(1,140) = 14.26, *p* < 0.001, η^2^ = 0.09; **Figure 1**]. Ascriptions of EA to advisors who recommended postponing the decision or taking out a loan of some sort were similar (all *F*s < 1.5, *p*s > 0.22).

Two-factor ANCOVA controlling for the average number of the correct answers in the manipulation check confirmed the significance of both main effects: type of advice, *F*(3,135) = 7.33, *p* < 0.001, η^2^ = 0.14, and experience, *F*(1,135) = 7.57, *p* = 0.007, η^2^ = 0.05 and the insignificance of the interaction between these two factors, *F*(3,135) = 0.34, *p* = 0.75. The effect of the covariant was not significant, *F*(3,135) = 0.26, *p* = 0.61. We also calculated the two-factor ANOVA only for participants who answered correctly to all items in the manipulation check and it confirmed the significance of both main effects, respectively *F*(3,80) = 6.184, *p* = 0.001, η^2^ = 0.19 for recommendation and *F*(1,80) = 7.57, *p* = 0.003, η^2^ = 0.10 for experience. The interaction between the two factors was again not significant, *F*(3,80) = 0.30, *p* = 0.82.

We also carried out regression analysis with advice as a dummy coded predictor and EA as the dependent variable. The advice recommending the greatest action (‘large loan’) was used as the reference point. There was an effect of ‘against’ advice, *b* = -0.73, *SE* = 0.19, *t* = -3.78, *p* < 0.001; but no effect of ‘postpone’ advice, *b* = -0.15, *SE* = 0.20, *t* = -0.73, *p* = 0.45, or ‘small loan’ advice, *b* = 0.10, *SE* = 0.20, *t* = 0.49, *p* = 0.62. This analysis confirmed that only the ‘against’ advice – recommending inaction – lowered the EA ascribed to a financial advisor; the other three forms of action advice’ all resulted in similar perceptions of the advisor’s EA.

The second main effect was related to experience. Lower EA was assigned to the advisor if he was described as having worked in a bank for 1 year (*M* = 3.18, *SD* = 0.93) rather than 15 years (*M* = 3.59, *SD* = 0.81).

### Discussion

The results of Experiment 1 suggest that lay people use various cues to evaluate financial experts’ authority, including experience. Not surprisingly, participants granted greater EA to more experienced financial advisors. This effect indicates that people do react reasonably to information when making judgments about expertise as greater experience is in many cases related to a deeper and more nuanced understanding (Klein, 1999, 2004). However, lay people show a sensitivity to cues which may result in evaluation biases. Similarly, to experiments in the field of judgments of medical expertise, we found that when evaluating financial expertise lay people tended to assign greater EA to consultants who recommended more action. It seems that a financial consultant who advises any form of action (in our scenario taking out some sort of loan, whether small or large) or at least contemplating action in the future (postponing the decision) is considered more expert than one who advises inaction.

To summarize, Experiment 1 provided preliminary evidence of bias in judgments of financial expertise: greater EA was ascribed to financial experts who advised greater action. We might speculate, however, that people who arrange to meet a financial advisor already have an opinion on the best course of action and that their evaluation of the expert may be influenced by the extent to which he or she endorses that opinion. For example, we may assume that when clients intentionally go to the bank to discuss the possibility of taking a loan they are already convinced that taking a loan is the best course of action available to them. If this was the case, they might be more likely to judge a consultant who recommends taking action as a valuable expert. This would suggest that the action advice effect found in Experiment 1 is only a specific manifestation of a broader phenomenon – the confirmation bias.

In Experiment 2 we added a new variable to capture participants’ opinions on financial activities and provide a preliminary test of the hypothesis that evaluation of EA is subject to a confirmation effect.

## Experiment 2: The Effects of Action Advice and Prior Opinion on Lay Evaluations of Experts’ Epistemic Authority

Experiment 2 further investigated the psychological nature of biases people commit in evaluating financial experts. We used the same loan scenario and the same four types of advice as in Experiment 1, but with two modifications. In Experiment 1 the client was described as having arranged to meet an advisor to talk about a loan, which may have led participants to assume that the client thought it would be a good idea to borrow the money to buy a flat. To examine this possibility we added a new variable to capture participants’ perception of the client’s opinion. If evaluations of EA were predicted by the interaction of the client’s pre-existing opinion with type of advice this would suggest that evaluations of financial expertise are subject to confirmation bias. We investigated whether participants would ascribe greater authority to experts whose advice was congruent with their perception of client’s *a priori* opinion on whether taking a loan is a proper choice.

### Method

#### Participants

We recruited 121 employed Polish adults having their own income (*M*_age_ = 35.39, *SD* = 6.17; 64 women). Participants were recruited on the streets and in two different academic institutions. Participants provided verbal consent to participation after the study had been described and it had been explained that participation was voluntary, that their data would be anonymous and that they could withdraw at any time. People who agreed to take part in the study met the interviewer at home or in another convenient setting (such as a university classroom) and completed the paper-and-pencil questionnaire individually. The study was voluntary, anonymous and in agreement with the guidelines of the Ethical Committee of the SWPS University of Social Sciences and Humanities, Faculty in Wroclaw. Participants did not receive any compensation. All materials and interactions with participants were in Polish language.

#### Procedure

Participants were randomly assigned to the one of four conditions in a between-subjects design, with type of advice as an independent variable.

After providing personal information (age; gender) participants were asked to read the hypothetical ‘bank loan’ scenario used in Experiment 1. We only manipulated type of advice and did not include any information about the advisor’s experience.

Participants read a scenario in which the consultant provided one of the four types of advice: (1) ‘against’ – the consultant advised the couple against taking out a mortgage and suggested that they should continue to rent; (2) ‘postpone’ – the consultant advised the couple to wait half a year and monitor the market; (3) ‘small action’ – the consultant advised taking out as small a loan as possible; (4) ‘large action’ – the consultant advised taking out a loan large enough to buy a flat and all the requisite home appliances.

#### Dependent Measures

After reading the scenario participants were asked to answer six questions assessing how much EA they attributed to the advisor (same as in Experiment 1, Cronbach’s α = 0.90).

#### Auxiliary Measures (Manipulation Check)

As in Experiment 1 participants responded to six additional items using a six-point scale ranging from 1 = ‘definitely disagree’ to 6 = ‘definitely agree.’ These questions were included as a manipulation check to verify that participants distinguished between the different types of an advice. Participants also answered one question evaluating their perception of the clients’ pre-existing opinion about taking out a loan (“What was the client’s opinion on taking loan held before the meeting”); responses were given on a seven-point scale ranging from 1 = ‘definitely negative’ to 7 = ‘definitely positive.’ The latter measure was used to preliminary test the possibility of the confirmation bias in evaluating the expert’s EA.

### Results

#### Manipulation Check

Before testing the main research hypotheses we performed a manipulation check based on MANOVA with the six additional items described above. As expected, we observed a main effect of advice, Wilks λ = 0.20, *F*(18,317) = 13.34, *p* < 0.001, η^2^ = 0.41. Further planned comparisons revealed that the participants were able to distinguish between the four types of advice, thus confirming the effectiveness of our experimental manipulation. Detailed results are presented in Supplementary Table S2. We again calculated a new variable indicating the number of correct answers to the manipulation check items, ranging from 0 – answers to all items were incorrect, to 6 – answers to all items were correct. None of the participants scored 0 on this variable, while 83.47% of them answered correctly to four or more items used as the manipulation check.

#### Advisor’s Epistemic Authority

One-factor ANOVA revealed a main effect of advice on perceived EA, *F*(3,117) = 5.48, *p* = 0.001, η^2^ = 0.12. Planned comparisons showed that the ‘against’ advice resulted in lower ratings of EA (*M* = 3.07, *SD* = 1.16) than the other three types of advice [‘postpone’: *M* = 3.85, *SD* = 1.01; *F*(1,117) = 7.86, *p* = 0.006, η^2^ = 0.06; ‘small loan’: *M* = 4.03, *SD* = 0.94; *F*(1,117) = 12.87, *p* < 0.001, η^2^ = 0.10; ‘large loan’: *M* = 3.96, *SD* = 1.00; *F*(1,117) = 11.64, *p* = 0.001, η^2^ = 0.09]. Ascriptions of EA in the three latter conditions were similar (all *F*s < 1, *p*s > 0.50); it seems that only the ‘against’ advice resulted in a lower EA rating for the financial advisor. This result confirms the Experiment 1 finding and shows that, in general lay people are highly reluctant to ascribe high EA to experts whose advice seems too cautious and who try to convince clients that their best course of action is to stick with the *status quo*.

The analysis of covariance (ANCOVA) controlling for the average number of the correct answers in the manipulation check confirmed the significance of both main effects: type of advice, *F*(3,116) = 5.49, *p* = 0.002, η^2^ = 0.12. The effect of the covariant was not significant, *F*(3,116) = 0.05, *p* = 0.82. We also calculated the one-factor ANOVA only for participants who answered correctly to all items in the manipulation check. It confirmed the significance of the main effect of recommendation, *F*(3,50) = 8.0, *p* < 0.001, η^2^ = 0.32.

#### Client’s *A priori* Opinion about Taking the Loan

We found no main effect of the type of advice on the perception of clients’ *a priori* opinion about taking the loan, *F*(3,117) = 1.37; *p* = 0.26. Insignificant effect for recommendations allows us to conclude that the participants – as instructed – indeed evaluated the client’s opinion on the bank loan that was held before meeting with an advisor, and not a modified opinion that might have been influenced by information obtained during this meeting. In general, participants believed that the client held positive opinions about the loan: one-sample *t*-test showed that the score was significantly higher than the middle point of the scale, *M* = 4.24, *SD* = 1.26, *t*(120) = 2.09, *p* = 0.038. Detailed results are presented in Supplementary Table S3.

#### The Effect of Type of Advice on EA Is Moderated by Client’s Prior Opinion on Loans

We examined whether the effect of type of advice on the advisor’s EA was moderated by the perceptions of the client’s prior opinion on loans. We carried out regression analysis with EA as the dependent variable and type of advice (dummy coded; ‘large loan’ advice as reference point), client opinion on loans and the interactions between these variables as predictors (all variables were z-scored for the regression). The overall model was significant, *F*(7,113) = 10.12, *p* < 0.001, *R*^2^ = 0.38. As in Experiment 1, only ‘against’ advice affected EA (‘against’: β = -0.35, *SE* = 0.09, *t* = -3.90, *p* = 0.001; ‘postpone’: β = -0.08, *SE* = 0.09, *t* = -0.87, *p* = 0.38; ‘small loan’: β = -0.02, *SE* = 0.09, *t* = -0.28, *p* = 0.78). Perceived client opinion also had an effect on perceptions of EA (β = 0.49, *SE* = 0.09, *t* = 5.71, *p* < 0.001). Most importantly, there was an interaction between client opinion and ‘against’ advice, β = -0.25, *SE* = 0.08, *t* = -3.12, *p* = 0.002, whilst the other interactions involving client opinion did not reach significance (‘postpone’: β = 0.05, *SE* = 0.12, *t* = 0.37, *p* = 0.71; ‘small loan’: β = -0.01, *SE* = 0.08, *t* = -0.08, *p* = 0.91).

**FIGURE 2:**
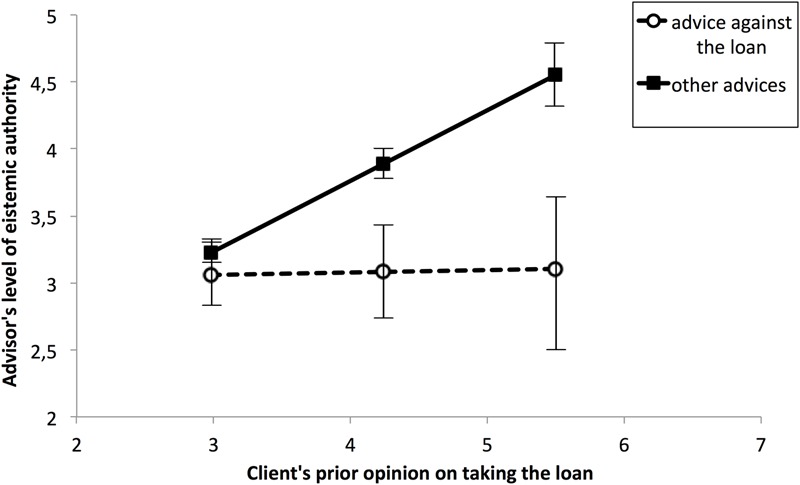
**Epistemic authority as a function of type of advice and perceptions of client’s prior opinion on loans**.

To decompose the above moderation effect we investigated how the relationship between ‘against’ advice and the dependent variable (advisor’s EA) differed from the relationship between other types of advice and EA at three levels of the moderator (client opinion): mean, 1 *SD* below the mean and 1 *SD* above the mean (respectively, raw score of 4.24, 2.98, and 5.5 on the seven-point scale; see **Figure 2**). ‘Against’ advice had no effect on EA at the lowest level of client opinion (β = -0.06, *SE* = 0.09, *t* = 0.70, *p* = 0.49), whereas at the intermediate and high levels it did (β = -0.32, *SE* = 0.07, *t* = -4.24, *p* < 0.001 and β = -0.57, *SE* = 0.11, *t* = -5.15, *p* < 0.001 respectively). As can be seen in **Figure 2**, when client opinion is perceived as neutral (scores around the midpoint of the scale) ratings of advisor EA are not affected by the type of advice they provide, but the more positive client opinion of loans, the stronger the conditional effect of type of advice on perceptions of EA; if the advisor advised against taking the loan when the client was perceived to favor doing he was ascribed a lower level of EA than when any other type of advice was given.

Next, we investigated this interaction using the Johnson–Neyman regions of significance (Hayes, 2013). This detailed analysis revealed that the effect of ‘against’ advice on EA was significant when the client opinion score was at least 3.45 out of 7 (i.e., when the client’s prior opinion on taking loans was perceived to be positive).

### Discussion

The results of Experiment 2 replicated the findings of Experiment 1 in that lay people tended to ascribe greater authority to experts who advised a more active approach. However, we once again found that there was a clear difference between the evaluations of advisors who advised against taking the loan and advisors who gave any other type of advice. We found that experts who advised against taking a loan were seen as less competent.

Experiment 2 showed, however, that the relationship between type of advice and evaluations of EA can also vary according to client opinion on the financial product in question. We found that lay people ascribed the greatest EA to the advisor in the scenario in which he advised taking action and where this coincided with the client’s perceived opinion on the advisability of taking out a loan. This result suggests that confirmation bias might contribute to evaluations of financial experts’ EA. However, there are two limitation of this conclusion. Firstly, in Experiment 2 the client’s opinion was measured, and not manipulated, what does not allow for causal conclusions. Secondly, we found that the average measured opinion toward a product was neutral or positive rather than negative. Experiment 3 was carried out to address these shortcomings in order to examine the confirmation bias effect more directly and in more detail.

## Experiment 3: Confirmation Bias in the Evaluation of Epistemic Authority of Financial Experts

To assess the role of confirmation bias in lay evaluations of financial expertise we decided to manipulate the hypothetical client’s opinion of various financial products directly. The participants were asked to imagine that their own opinion on the whether to accept the financial product was positive or negative. Our expectation was that if lay EA evaluations were affected by confirmation bias providing action advice would only enhance an advisor’s EA if it coincided with client’s prior opinion (i.e., the client was in favor of the proposed course of action). If the client’s prior opinion was against taking out a loan we predicted that action advice would have no effect on evaluations of the advisor’s EA.

Another modification was related to the stimuli used in the experiment. In Experiments 1 and 2 we used a bank loan as an example of a financial product. In this experiment we used another product (life insurance policy) to test the robustness of the effects we had found earlier.

### Method

#### Participants

We recruited 112 employed Polish adults having their own income (*M*_age_ = 26.37, *SD* = 6.41; 94 women). Participation in the study was voluntary and participants did not receive any compensation. The experiment was conducted online and participants were recruited via open groups on Facebook and various social forums. The study was voluntary, anonymous and in agreement with the guidelines of the Ethical Committee of the SWPS University of Social Sciences and Humanities, Faculty in Wroclaw. Participants did not receive any compensation. All materials and interactions with participants were in Polish language.

#### Procedure

Participants were randomly assigned to the one of four groups in a between-subjects factorial design, with type of advice (two levels: ‘against’; ‘for’) and client’s prior opinion (two levels: negative; positive) as independent variables. After they had given informed consent and provided personal information participants were asked to read a hypothetical scenario concerning the purchase of life insurance. Participants were asked to imagine that they had attended a routine meeting with the insurance agent and that during the meeting the discussion had turned to life insurance. They were then presented with the consultant’s advice, accompanied by a very short justification. In this experiment only two advice conditions were used: ‘against’ life insurance (advice not to buy) and ‘for’ life insurance (advice to buy). We limited the number of conditions, because Experiments 1 and 2 showed that people only differentiated between extreme inaction advice and all other forms of advice when evaluating the advisor’s EA. Moreover, using two levels of advice manipulation allowed for a more effective examination of the confirmation bias effect. In the ‘against’ condition the consultant advised against buying the life insurance product as it was quite expensive whereas in the ‘pro’ condition the consultant advised buying the insurance product as it was quite cheap.

The consultant’s advice was followed by a brief description of the participant’s opinion. In the ‘positive’ condition participants were asked to imagine that they were interested in this kind of insurance, because one of their friends who had already purchased it said that it was very profitable. In the ‘negative’ condition participants were asked to imagine that they were not convinced that life insurance was worthwhile because their friend who had already purchased it said that it was very unprofitable.

#### Dependent Measure

After reading the scenario participants were asked to answer the same six questions assessing EA as in the two previous experiments (Cronbach’s α = 0.91).

### Results

**FIGURE 3:**
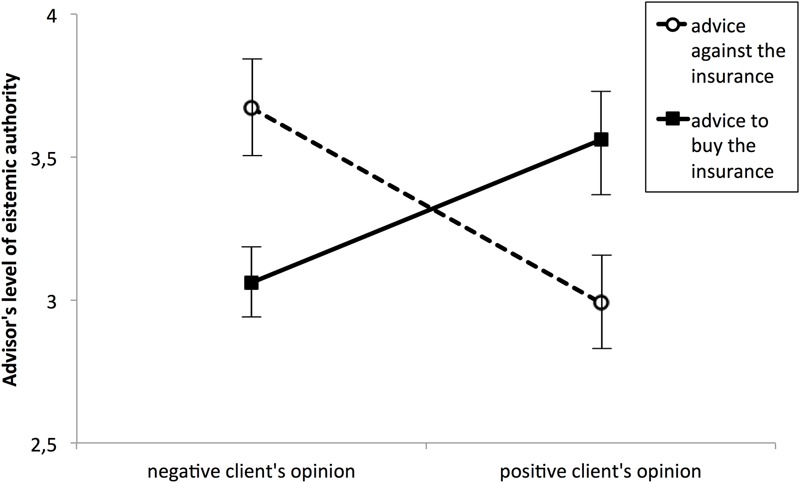
**Perceived epistemic authority of advisors as a function of their advice and client opinion on purchasing life insurance**.

A two (type of advice: against; for) by two (client opinion: positive; negative) ANOVA with advisor’s EA as the dependent variable was used to test the main hypothesis. There was no main effect of type of advice, *F*(1,108) = 0.03, *p* = 0.86, or client opinion, *F*(1,108) = 0.373, *p* = 0.543. As expected, the two independent factors interacted with each other, *F*(1,108) = 14.118, *p* < 0.001, η^2^ = 0.116 (see **Figure 3**).

Further planned comparisons revealed that when client opinion of life insurance products was positive the participants ascribed greater EA to advisors who recommended purchasing it (*M* = 3.56, *SD* = 0.87) than to those who recommended rejecting it [*M* = 2.99, *SD* = 0.84; *F*(1,108) = 5.99, *p* = 0.016, η^2^ = 0.05]. However, when client opinion was negative the reverse pattern applied, ‘against’ advice was associated with higher EA ratings (*M* = 3.68, *SD* = 0.89) than the ‘for’ advice [*M* = 3.06, *SD* = 0.70; *F*(1,108) = 8.33, *p* = 0.005, η^2^ = 0.07].

### Discussion

Experiment 3 supported the hypothesis that lay evaluations of the financial experts’ EA are affected by confirmation bias. The participants tended to ascribe greater EA to advisors whose advice was congruent with the client’s prior opinion. This result also suggests that there are limits to the action advice effect demonstrated in Experiments 1 and 2. It seems that lay people tend to ascribe greater EA to financial consultants who give action advice (e.g., to purchase a financial product) only if this advice is consistent with their own opinion (e.g., they also think that purchasing the product in question is a good idea).

## General Discussion

The most important aim of this project was to examine how lay people who do not possess a professional knowledge in economics evaluate financial consultants differing in their advice. In particular, the three experiments presented in this paper tested how the two psychological effects – the effect of an action advice and the confirmation bias – might contribute to our understanding of lay evaluation of EA in the field of finance.

Experiments 1 and 2 showed that the action advice effect that favors physicians advising aggressive treatment (Barnoy et al., 2012; Bar-Tal et al., 2013; Stasiuk et al., 2016) might also hold in the financial field. Data collected in these two experiments revealed that financial consultants who advised against taking out a bank loan (i.e., who advised inaction) received worse EA evaluations than those who suggested a less inactive approach (i.e., at the very least recommended postponing the decision or advised taking out a loan immediately). However, it cannot be excluded that clients who arrange to meet a financial advisor already have a positive opinion on a more active course of action and that their evaluation of the expert may be influenced by the extent to which he or she advices action. If this was the case, they might be more likely to judge a consultant who recommends taking action as a valuable expert. This interpretation suggests that the action advice effect found in Experiment 1 might have been only a specific manifestation of a broader phenomenon – the confirmation bias.

Indeed, the outcomes of Experiment 2 and, particularly, Experiment 3 suggested that the action advice effect might result from clients’ desire to confirm their own opinions. In Experiment 3 participants only ascribed greater authority to an advisor who recommended accepting the financial product in question (life insurance) when they held a positive opinion of the product. Participants with a negative opinion of the product tended to ascribe greater EA to an advisor who advised rejecting it. This interaction between type of advice and client opinion suggests that there are limits to the action advice effect described earlier. The action advice effect seems to influence lay evaluations only when the people’s opinions or preferences are ambiguous or not salient. Another possibility is that relying on the action advice cue in evaluations of EA is simply another reflection of confirmation bias: when lay people are uncertain of their own beliefs, they try to confirm a common belief that experts should advise action rather than maintenance of the *status quo*. This possibility warrants further research.

Why are people prone not only to the action advice effect but also to confirmation bias when evaluating financial advisors’ EA? Nickerson (1998, p. 175) argues that confirmation bias “connotes the seeking or interpreting of evidence in ways that are partial to existing beliefs, expectations, or a hypothesis in hand.” This definition suggests that confirmation bias amounts to a selective search for information and discrimination in the use of it. In other words, it is described as a purely cognitive inclination. However, confirmation bias might also be understood as a part of the broader phenomenon of ‘motivated reasoning’ (Kunda, 1987, 1990; Molden and Higgins, 2005; Mercier and Sperber, 2011). Research has shown that people engage in ‘motivated thinking’ to defend their beliefs and to preserve a positive view of themselves (Mercier and Sperber, 2011) or to minimize negative and maximize positive affective states (Westen et al., 2006). Even if lay people do not have an expert knowledge of a given area and hence feel that they have to turn to an expert for advice, it does not necessarily mean that they do not have opinions or beliefs on issues in the area. Lay people often hold naïve theories of reality (e.g., economic reality; see Furnham and Lewis, 1986; Lea et al., 1987) that help them to structure and understand the world surrounding them. One reason for consulting an expert might be to confirm a personal theory or belief. For example, if an individual thinks that investing in stocks is a good long-term saving strategy then receiving advice which supports this opinion from a consultant would be not only help him or her to make a decision about how to invest but would also be psychologically rewarding. If, however, an expert provides advice which contradicts a lay person’s beliefs that person might try to devalue or discredit the advice by underestimating the authority of the expert who provided it in order to shore up their prior position. Evaluation of experts’ EA would thus be influenced by the basic drive to reduce cognitive dissonance (Festinger, 1957). Further research should be conducted to examine this possibility in more depth. Future experiments should focus on what happens when people’s opinions are ambiguous or implicit. We predict that in these circumstances lay people’s evaluations of experts’ EA might be subject to a bias to confirm various external sources of knowledge (e.g., common beliefs, opinions of a trusted individual).

Another issue that warrants exploration in future experiments is the role of people’s own financial knowledge. The authors of lay epistemic theory introduced the concept of self-epistemic authority (SEA) to capture individuals’ perceptions of their own competence in a given field (Ellis and Kruglanski, 1992; Raviv et al., 1992). It seems plausible that financial SEA would influence perceptions of the EA of financial experts. We hypothesize that an individual with high SEA would find the endorsement of her opinion by an expert validating and would ascribe higher EA to an expert whose advice is compatible with her opinions. For individuals with low SEA, however, having one’s opinion endorsed by an expert would evoke ambivalent feelings and hence low SEA individuals might ascribe a lower EA to this expert (Kruglanski et al., 2005).

Do the results of this study mean that people only use superficial cues when evaluating the authority of financial experts and that their evaluations will inevitably be biased? Experiment 1 suggested that people might also base their judgments on more objective sources of information. Our participants EA ratings suggested that they believed that greater weight should be given to the opinion of more experienced advisors. However, this experiment also showed that lay peoples’ evaluations of financial experts could be dominated by superficial rather than objective cues. Data analysis revealed that the EA evaluations were more strongly influenced by recommendation than experience. Lay people typically turn to experts for help in making better, more thorough decisions; however, our three experiments showed that when selecting a financial advisor the need to receive an advice for action or the desire to confirm one’s own beliefs might prevail over the need for objective information about the quality of financial advice.

The current experimental project certainly holds its limitations. Firstly, all three experiments used only hypothetical scenarios presenting the interaction between a lay person and a financial advisor. Moreover, the participants were asked to evaluate an advisor without being requested to make even hypothetical decisions. Secondly, the participants were not incentivized. In our future research we are going to study judgments and choices in more naturalistic situations in which people will have a chance to either accept or reject advisor’s recommendations when making their decisions. Using real incentives will allow us to test whether the results found in the present project hold when the subject’s own money is at stake. Certainly, an ideal solution would be to conduct field experiments in which we might study behaviors of customers making real financial decisions and taking advice from real financial experts.

The results presented in this paper have important practical as well as theoretical implications. Engelmann et al. (2009) reported that financial advice might ‘oﬄoad’ people’s own opinions when making decisions about risk-taking. These authors found that when expert advice was available, lay people tended to make choices following it. Moreover, neurophysiological data showed that neural activation was not correlated with evaluations of options with different levels of risk in the presence of an expert recommendation. Later research by Engelmann et al. (2012) also showed that the behavioral impact of the advice is likely influenced by the amount of personal experience with the type of decision that the advice is related to. These authors report that the enhanced effect of advice from an expert economist on risky financial decisions was observed in younger adolescents, compared to adults who typically have more experience in making financial choices under risk. This implies that lay people, and especially those whose experience is extremely poor, might rely unthinkingly on professional advice without carefully analyzing accessible alternatives. This importance of such an effect is clear when it is linked with our results showing that lay evaluations of expert advice are biased. Lay decision-makers who use financial advice and evaluate its source as highly competent should consider the real basis for that evaluation. One recommendation that could be made to consumers of financial advice on the basis of the results presented here is that they should consider both advice that is compatible with their beliefs and advice that is not in order to minimize their vulnerability to bias.

## Author Contributions

All authors developed the conception of work and design of experiments. TZ, AG, KS, and RM collected the data; TZ and AG analyzed the data, and all authors interpreted the data. TZ and AG drafted the manuscript; YB-T, KS, and RM provided critical revisions. All authors approved the final version of the manuscript for submission.

## Conflict of Interest Statement

The authors declare that the research was conducted in the absence of any commercial or financial relationships that could be construed as a potential conflict of interest.

## Footnotes

1 In all experiments the financial advisors were men to avoid confounding effects of advisor gender, we therefore use the ‘he’ pronoun when describing experimental scenarios.
