# Supplementary material for: Lay Evaluation of Financial Experts: The Action Advice Effect and Confirmation Bias
Source: Front Psychol. 2016 Sep 27;7:1476. doi: 10.3389/fpsyg.2016.01476 (PMC5037174; doi:10.3389/fpsyg.2016.01476)
Supplement: Supplementary file 1 [file Table_1.DOCX]

Supplementary table 1. Experiment 1 – detailed results of the manipulation check

| Dependent Variable | Advice | | | | | | | |  |  |  |
| --- | --- | --- | --- | --- | --- | --- | --- | --- | --- | --- | --- |
|  | against | | postpone | | small advice | | big advice | | *F*(3, 140) | *p* | η^2^ |
| Advisor was against taking the loan | 4.500 | ^a^ | 4.059 | ^a^ | 1.941 | ^b^ | 1.500 | ^b^ | 59.239 | .001 | .559 |
|  | (0.191) |  | (0.202) |  | (0.202) |  | (0.191) |  |  |  |  |
| Advisor was opting for taking the loan | 2.000 | ^a^ | 1.735 | ^a^ | 4.500 | ^b^ | 5.158 | ^b^ | 85.662 | .001 | .647 |
|  | (0.183) |  | (0.193) |  | (0.193) |  | (0.183) |  |  |  |  |
| Advisor's opinion on the loan was definitely negative | 4.237 | ^a^ | 3.706 | ^a^ | 2.147 | ^b^ | 2.053 | ^b^ | 30.564 | .001 | .396 |
|  | (0.196) |  | (0.207) |  | (0.207) |  | (0.196) |  |  |  |  |
| Advisor's opinion on the loan was definitely positive | 2.158 | ^a^ | 2.471 | ^a^ | 4.176 | ^b^ | 4.237 | ^b^ | 30.633 | .001 | .396 |
|  | (0.195) |  | (0.206) |  | (0.206) |  | (0.195) |  |  |  |  |
| Advisor's advise was to take a large loan | 2.053 | ^a, c^ | 1.618 | ^a, b^ | 2.235 | ^b, c^ | 5.000 |  | 54.864 | .001 | .540 |
|  | (0.205) |  | (0.217) |  | (0.217) |  | (0.205) |  |  |  |  |
| Advisor's advise was to take a small loan | 1.974 | ^a, b^ | 1.882 | ^a, c^ | 4.588 |  | 1.711 | ^b, c^ | 59.787 | .001 | .562 |
|  | (0.169) |  | (0.179) |  | (0.179) |  | (0.169) |  |  |  |  |

Standard errors in parentheses. Pairwise comparisons with least significant difference adjustment for multiple comparisons. Same indexes in each row indicates the means that are not significantly different at p<0.01
