# Supplementary material for: Lay Evaluation of Financial Experts: The Action Advice Effect and Confirmation Bias
Source: Front Psychol. 2016 Sep 27;7:1476. doi: 10.3389/fpsyg.2016.01476 (PMC5037174; doi:10.3389/fpsyg.2016.01476)
Supplement: Supplementary file 2 [file Table_2.DOCX]

Supplementary table 2. Experiment 2 – detailed results of the manipulation check

| Dependent Variable | Advice | | | | | | | |  |  |  |
| --- | --- | --- | --- | --- | --- | --- | --- | --- | --- | --- | --- |
|  | against | | postpone | | small advice | | big advice | | *F*(3, 117) | *p* | η^2^ |
| Advisor was against taking the loan | 4.552 | ^a^ | 3.593 | ^a^ | 2.452 | ^b^ | 1.676 | ^b^ | 21.029 | .001 | .350 |
|  | (0.285) |  | (0.295) |  | (0.275) |  | (0.263) |  |  |  |  |
| Advisor was opting for taking the loan | 1.966 | ^a^ | 2.815 | ^a^ | 4.484 | ^b^ | 5.176 | ^b^ | 34.220 | .001 | .467 |
|  | (0.260) |  | (0.270) |  | (0.252) |  | (0.240) |  |  |  |  |
| Advisor's opinion on the loan was definitely negative | 4.655 |  | 3.037 | ^a^ | 2.355 | ^ab^ | 1.794 | ^b^ | 22.838 | .001 | .369 |
|  | (0.266) |  | (0.276) |  | (0.257) |  | (0.246) |  |  |  |  |
| Advisor's opinion on the loan was definitely positive | 1.966 |  | 3.148 |  | 4.419 | ^a^ | 4.853 | ^a^ | 24.216 | .001 | .383 |
|  | (0.273) |  | (0.283) |  | (0.264) |  | (0.252) |  |  |  |  |
| Advisor's advise was to take a large loan | 1.552 | ^a, b^ | 2.037 | ^a, c^ | 2.306 | ^b, c^ | 5.015 |  | 43.417 | .001 | .527 |
|  | (0.250) |  | (0.259) |  | (0.241) |  | (0.230) |  |  |  |  |
| Advisor's advise was to take a small loan | 1.621 | ^a^ | 2.630 | ^b^ | 4.581 |  | 1.706 | ^a, b^ | 28.719 | .001 | .424 |
|  | (0.266) |  | (0.275) |  | (0.257) |  | (0.245) |  |  |  |  |

Standard errors in parentheses. Pairwise comparisons with least significant difference adjustment for multiple comparisons. Same indexes in each row indicates the means that are not significantly different at p<0.01
