# Supplementary material for: Lay Evaluation of Financial Experts: The Action Advice Effect and Confirmation Bias
Source: Front Psychol. 2016 Sep 27;7:1476. doi: 10.3389/fpsyg.2016.01476 (PMC5037174; doi:10.3389/fpsyg.2016.01476)
Supplement: Supplementary file 3 [file Table_3.DOCX]

Supplementary table 3. Experiment 2 – detailed results of the client’s opinion measure

|  |  | recommendation | | | | Total |
| --- | --- | --- | --- | --- | --- | --- |
| what was the client's opinion on taking loan before the meeting | | against | postpone | small loan | big loan |  |
| 1 | Count | 1 | 0 | 1 | 1 | 3 |
| (definitelly negative) | % within condition | 3,40% | 0,00% | 3,20% | 2,90% | 2,50% |
| 2 | Count | 3 | 0 | 2 | 2 | 7 |
| (negative) | % within condition | 10,30% | 0,00% | 6,50% | 5,90% | 5,80% |
| 3 | Count | 7 | 2 | 1 | 7 | 17 |
| (rather negative) | % within condition | 24,10% | 7,40% | 3,20% | 20,60% | 14,00% |
| 4 | Count | 11 | 15 | 12 | 10 | 48 |
| (not negative, nor positive) | % within condition | 37,90% | 55,60% | 38,70% | 29,40% | 39,70% |
| 5 | Count | 4 | 8 | 8 | 8 | 28 |
| (rather positive) | % within condition | 13,80% | 29,60% | 25,80% | 23,50% | 23,10% |
| 6 | Count | 1 | 2 | 6 | 4 | 13 |
| (positive) | % within condition | 3,40% | 7,40% | 19,40% | 11,80% | 10,70% |
| 7 | Count | 2 | 0 | 1 | 2 | 5 |
| (difinitelly positive) | % within condition | 6,90% | 0,00% | 3,20% | 5,90% | 4,10% |
| Total | Count | 29 | 27 | 31 | 34 | 121 |
|  | % within condition | 100,00% | 100,00% | 100,00% | 100,00% | 100,00% |
